# Effect of Eucalyptus Oil Inhalation on Pain and Inflammatory Responses after Total Knee Replacement: A Randomized Clinical Trial

**DOI:** 10.1155/2013/502727

**Published:** 2013-06-18

**Authors:** Yang Suk Jun, Purum Kang, Sun Seek Min, Jeong-Min Lee, Hyo-Keun Kim, Geun Hee Seol

**Affiliations:** ^1^Department of Basic Nursing Science, School of Nursing, Korea University, Anam-dong, Seongbuk-gu, Seoul 136-713, Republic of Korea; ^2^Department of Physiology and Biophysics, School of Medicine, Eulji University, Daejeon 301-746, Republic of Korea; ^3^KT&G Research Institute, Daejeon 305-805, Republic of Korea

## Abstract

Eucalyptus oil has been reported effective in reducing pain, swelling, and inflammation. This study aimed to investigate the effects of eucalyptus oil inhalation on pain and inflammatory responses after total knee replacement (TKR) surgery. Participants were randomized 1 : 1 to intervention group (eucalyptus inhalation group) or control group (almond oil inhalation group). Patients inhaled eucalyptus or almond oil for 30 min of continuous passive motion (CPM) on 3 consecutive days. Pain on a visual analog scale (VAS), blood pressure, heart rate, C-reactive protein (CRP) concentration, and white blood cell (WBC) count were measured before and after inhalation. Pain VAS on all three days (*P* < .001) and systolic (*P* < .05) and diastolic (*P* = .03) blood pressure on the second day were significantly lower in the group inhaling eucalyptus than that inhaling almond oil. Heart rate, CRP, and WBC, however, did not differ significantly in the two groups. In conclusion, inhalation of eucalyptus oil was effective in decreasing patient's pain and blood pressure following TKR, suggesting that eucalyptus oil inhalation may be a nursing intervention for the relief of pain after TKR.

## 1. Introduction

Osteoarthritis, the most prevalent musculoskeletal disorder throughout the world, is a common chronic disease that causes pain, restricts activity, and reduces quality of life [1]. Osteoarthritis may occur in all joints, but the knee is the most frequent site [2]. The most common clinical features of osteoarthritis include pain, stiffness, swelling, and inflammation. Surgery may be considered in patients who do not show symptom improvements on nonsurgical treatments, especially when severe pain interferes with daily life [3]. Total knee replacement (TKR) is a surgical procedure in which deformed knee cartilage is resected and replaced by a metal structure filled with polyethylene, resulting in a new joint structure. TKR has been shown to improve the quality of life of patients with severe arthritis by relieving knee pain and increasing knee function [4]. Inflammation by infection after TKR has a negative impact on patient prognosis, with deep infection requiring a second operation [5]. Thus, inflammation control as well as pain management are required for rapid recovery and functionality. Eucalyptus (*Eucalyptus globulus*) oil has been widely used as a folk medicine to treat upper respiratory infections, gastritis, and diabetes [6].

Eucalyptus oil contains *α*-pinene and 1,8-cineole and acts as an antioxidant, with strong radical scavenging activity [7]. In a mouse model of pain-causing edema in the feet, oral administration of 1,8-cineole, which accounts for 70–90% (w/w) of the contents of eucalyptus oil, suppressed edema formation and reduced inflammation and pain [8]. This effect of 1,8-cineole is due to its inhibition of cytokine secretion by T-lymphocytes [9]. Electromyography has shown that application of eucalyptus oil to a healthy subject had a myorelaxant effect, as well as promoting emotional stability [10]. Moreover, in a rat model of susceptibility to pain from a hot plate, eucalyptus oil was not only analgesic but reduced edema formation and had an anti-inflammatory effect [11].

Although eucalyptus oil has been found to reduce pain and suppress edema and inflammation in animal models, its effects on pain in patients who have undergone TKR have not been determined. We therefore assessed whether eucalyptus oil inhalation could effectively reduce pain and inflammatory responses in patients who have undergone TKR.

## 2. Materials and Methods

### 2.1. Study Design and Sample Size

Patients were randomly assigned using table of random numbers to inhalation of eucalyptus oil or almond oil (used as solvent, control) during continuous passive motion (CPM) after TKR, with both investigators and subjects blinded to treatment assignment. Based on an effect size of 0.80, a statistical power of 0.80, and a significance level of 0.05, the minimum number of patients required to compare differences between the experimental and control group was estimated to be 26 patients per group. Twenty-eight subjects were originally assigned to each group, with 52 completing the study, 25 in the experimental and 27 in the control group.

### 2.2. Participants

Patients diagnosed with osteoarthritis by the same physician and who underwent TKR were invited to participate. All subjects (1) had been prescribed pain medications, including oxycodone hydrochloride, fentanyl, nonsteroidal anti-inflammatory drugs (NASID), and antibiotic pills, (2) had no complications or other inflammatory diseases after surgery, (3) were not being treated with any antidepressant, hormone, or aroma therapy, (4) did not smoke, (5) were conscious and oriented, and (6) had a pain score on a visual analog scale (VAS) >4 before aroma inhalation. The study design and protocol were approved by the Ethical Review Committee of the Korea University Medical Center (Code: ED11285), and all participants provided written informed consent.

### 2.3. Gas Chromatography-Mass Spectrometry Analysis

Gas chromatography-mass spectrometry (GC-MS) was performed to analyze compositions of eucalyptus oil. GC-MS analysis was performed using an Agilent 7890 gas chromatograph with a 5975 inert mass spectrometer (USA), and analytical capillary column was performed on HP-Innowax (60 m × 0.25 mm i.d., 0.5 *μ*m film thickness, Agilent, USA). Carrier gas was helium and flowrate was 1.0 mL/min. The injector temperature was increased to 280°C. And samples (1.0 *μ*L) were injected at a split ratio of 50 : 1. The column temperature was initially maintained at 50°C (hold for 3 min), ramp to 240°C at 3°C/min, and finally held for 10 min. The ion source and transfer line temperature were set at 230°C and 250°C. The mass spectrometer was operated in the electron impact ionization mode (70 eV).

### 2.4. Intervention

Eucalyptus oil and almond oil were supplied by Aromarant Co. Ltd. (Rottingen, Germany). Eucalyptus oil was dissolved at a concentration of 3% (v/v) in almond oil. In experimental group, eucalyptus oil (dissolved in almond oil) was placed onto a 4 × 2 inch gauze pad, which was positioned between the nose and philtrum for 30 minutes of CPM on 3 consecutive days, beginning on the third day after surgery. The control group received the same treatment of experimental group except for eucalyptus. Eucalyptus oil and almond oil have a similar color and were packed same-shaped bottles. All experiments were carried out separately. The compounder was the only one who knows which participant affiliated to which group according to the assigned number on bottle. Patients and investigator were not informed about the types and effects of aroma oil.

### 2.5. Outcome Measurement

Pain was measured using the VAS pain score. It is also used in most studies of anxiety, consists of a horizontal scale, ranging from 0 (no pain) on the left side and 10 (extreme pain) on the right side [12]. Patients were asked to indicate VAS pain score by number at each determination. Blood pressure and heart rate were measured on each day of oil inhalation, before and after CPM, as indicators of the reaction of the autonomic nervous system to pain. Blood pressure was measured in the brachial artery using a Deluxe Aneroid Sphygmomanometer (Mac-check, Japan) after a 10-minute rest in a lying position. Pulse was measured at the radial artery for 1 minute.

In this study, CRP was measured by a Latex Immunoassay with LX-2200 (Eiken Inc., Japan) and WBC was measured by semiconductor flow cytometry with an XE-5000 (Sysmex, Inc., Japan).

### 2.6. Data Collection

Blood samples were collected before breakfast on the first day of aroma inhalation and on days 4 and 7 for measurements of C-reactive protein (CRP) concentrations and white blood cell (WBC) count. Pain VAS, blood pressure, and heart rate were measured before and after each 30 min inhalation session.

### 2.7. Statistical Analysis

All statistical analyses were performed using the Statistical Package for the Social Sciences, SPSS 20.0. The demographic and clinical characteristics of the two groups were compared using *t*-tests, Fisher's exact tests, and Chi-square tests, as appropriate. All data are presented as frequency, percentages, and standard error of the mean (SEM) or standard deviation (SD). Differences in the VAS pain scores before and after aroma inhalation within each group were compared using the Wilcoxon's rank-sum test and paired *t*-test, and differences between groups were analyzed using Mann-Whitney *U*-tests and unpaired *t*-tests.

## 3. Results

### 3.1. Compositions of Eucalyptus Oil

A total of 31 compounds were identified (Table 1). The major volatile flavor compounds of eucalyptus oil were 1,8-cienol (61.46%), limonene (13.68%), *ρ*-cymene (8.55%), *γ*-terpinene (5.87%), and *α*-pinene (4.95%).

### 3.2. Characteristics of the Participants and Test of Homogeneity

Fifty-two individuals were randomized, 25 to the eucalyptus oil and 27 to the almond oil (control) group. Demographic and disease-associated characteristics were similar in the two groups, including type of surgery, duration of osteoarthritis, and treatments with oral antihypertensive and antidiabetic agents (Table 2). The mean age of the 52 participants was 68.2 years (range, 43–85 years), and most were female. Their average body mass index (BMI) was 26.4 ± 3.1 kg/m^2^, and all 52 were overweight/obese (BMI ≥ 25 kg/m^2^). Thirty-seven patients (71.2%) underwent a unilateral TKR and 15 (28.9%) underwent a simultaneous, bilateral TKR. The mean durations of osteoarthritis in the eucalyptus and almond oil groups were 8.5 ± 5.7 years and 6.0 ± 5.2 years, respectively. Before aroma inhalation, VAS pain score, systolic blood pressure, diastolic blood pressure, heart rate, CRP, and WBC count were similar in the two groups (Table 3).

### 3.3. Effects of Eucalyptus Oil on VAS Pain Score

**Figure 1:**
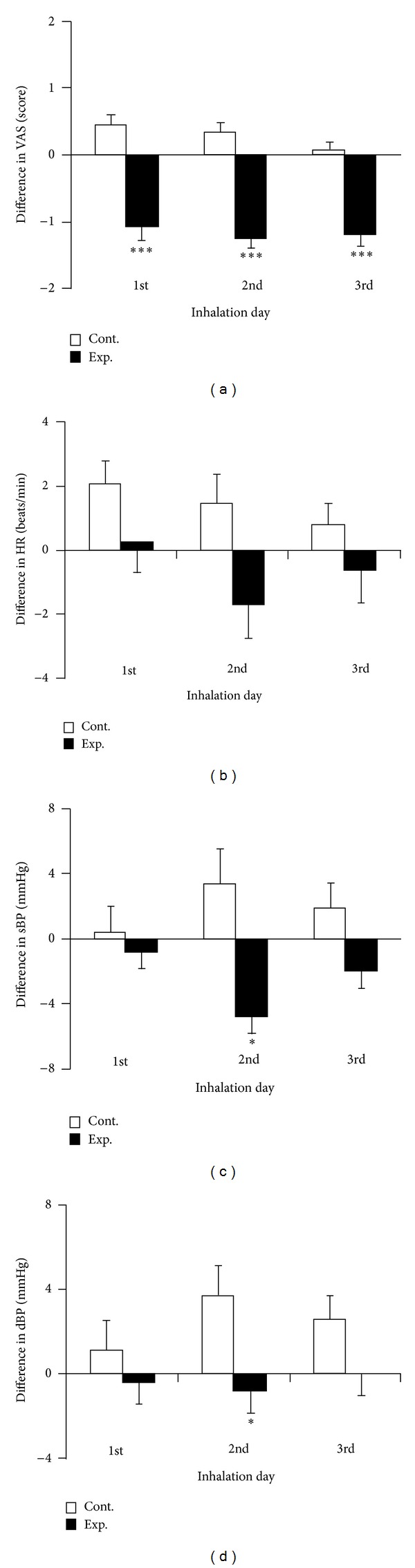
Effects of inhalation on (a) VAS, (b) HR, (c) sBP, and (d) dBP in the eucalyptus oil (*n* = 25) and control (almond oil; *n* = 27) groups. Results are expressed as mean ± SEM. *P < .05, ***P < .001 compared with the control group. Abbreviations: VAS, visual analog scale; HR, heart rate; sBP, systolic blood pressure; dBP, diastolic blood pressure.

VAS pain scores after aromatherapy on days 1–3 decreased 1.1 ± 0.2, 1.2 ± 0.2, and 1.2 ± 0.2 points, respectively, from the scores before inhalation (Figure 1(a)). In the control group, however, VAS pain scores on days 1–3 increased 0.4 ± 0.2, 0.3 ± 0.2, and 0.1 ± 0.1 points, respectively, from the scores before inhalation. Overall, VAS pain scores were significantly lower in the eucalyptus oil group than in the control group (*P* < .001, Figure 1(a)).

### 3.4. Effects of Eucalyptus Oil on Heart Rate and Blood Pressure

Relative to pretreatment heart rate, heart rate in the eucalyptus oil group increased 0.3 ± 1.6 beats/min on day 1 of CPM and decreased 1.7 ± 1.7 beats/min and 0.6 ± 1.0 beats/min on days 2 and 3, respectively (Figure 1(b)). The heart rate in the control group, however, showed increases after CPM of 2.1 ± 0.7, 1.5 ± 0.9, and 0.8 ± 0.7 beats/min on days 1–3 of CPM, respectively. Between-group differences in heart rate did not differ significantly.

Systolic blood pressure on days 1–3 decreased 0.8 ± 1.9 mmHg, 4.8 ± 2.2 mmHg, and 2.0 ± 2.0 mmHg, respectively, in the eucalyptus oil group, while increasing 0.4 ± 1.7 mmHg, 3.3 ± 2.2  mmHg, and 1.9 ± 1.6 mmHg, respectively, in the control group (Figure 1(c) ). On day 2, SPB was significantly lower in the eucalyptus oil than in the control group (*P* < .05, Figure 1(c)). Similarly, diastolic blood pressure in the eucalyptus oil group decreased 0.4 ± 1.5 mmHg, 0.8 ± 1.5 mmHg, and 0.0 ± 1.2 mmHg, respectively, on days 1–3, while increasing 1.1 ± 1.5 mmHg, 3.7 ± 1.4 mmHg, and 2.6 ± 1.1 mmHg, respectively, in the control group on the same days. Diastolic blood pressure on day 2 was significantly lower in the eucalyptus oil group than in the control group (*P* = .03, Figure 1(d)).

### 3.5. Effects of Eucalyptus Oil on Inflammatory Responses

**Figure 2:**
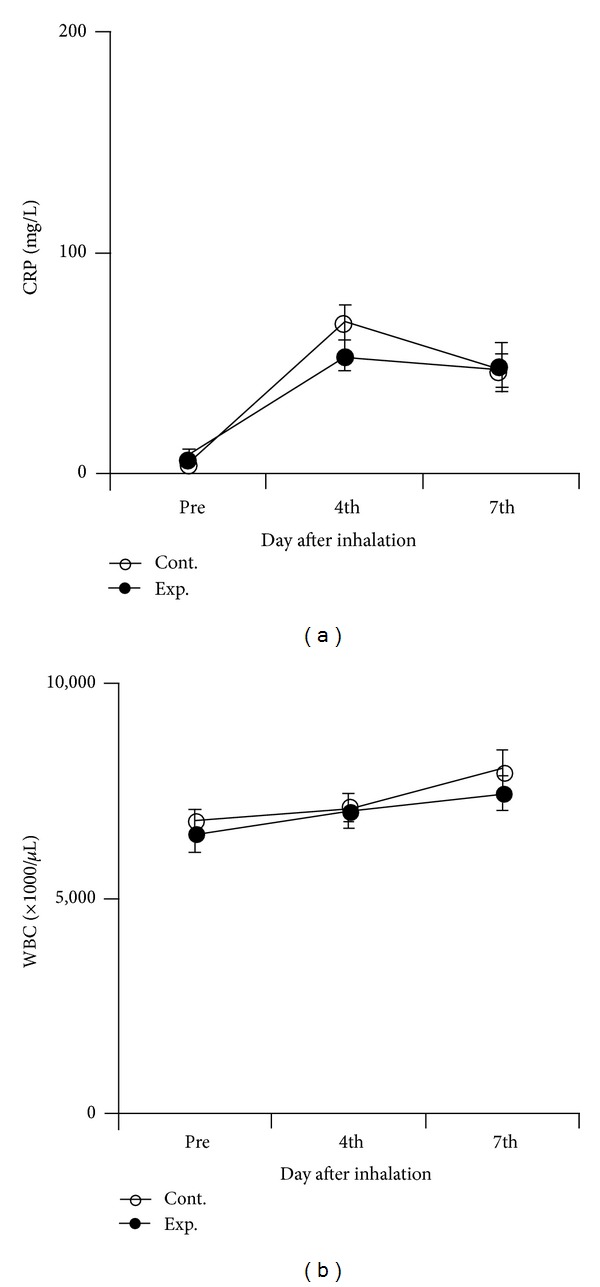
Effects of inhalation on (a) CRP and (b) WBC in the eucalyptus oil (*n* = 25) and control (almond oil; *n* = 27) groups. Values are expressed as mean ± SEM. Abbreviations: CRP, C-reactive protein; WBC, white blood cell.

Serum CRP concentrations before inhalation and on days 4 and 7 were 7.2 ± 3.9, 53.5 ± 6.8, and 48.8 ± 11.0 mg/L, respectively, in the eucalyptus oil group, and 4.89 ± 2.0, 68.2 ± 8.2, and 46.8 ± 7.7 mg/L, respectively, in the control group (Figure 2(a)). Although CRP concentrations in both groups tended to increase gradually after surgery and then decrease, no between-group significant differences were observed.

WBC counts before inhalation and on days 4 and 7 were 6, 513.2 ± 417.0 × 10^3^/*μ*L, 7,062.0 ± 377.9 × 10^3^/*μ*L, and 7,450.0 ± 383.5 × 10^3^/*μ*L, respectively, in the eucalyptus oil group, and 6,793.3 ± 268.8 × 10^3^/*μ*L, 7, 112.6 ± 336.9 × 10^3^/*μ*L, and 7,970 ± 502.4 × 10^3^/*μ*L, respectively, in the control group (Figure 2(b)). None of these differences reached statistical significance.

## 4. Discussion

Since eucalyptus oil has been reported effective in reducing pain and suppressing inflammation in the various animal models, we tested whether inhalation of eucalyptus oil affected pain, blood pressure, heart rate, CRP concentration, and WBC count following TKR in patients with osteoarthritis.

Pain is an emotion which is quintessentially subjective and personal [13, 14]. To assess patient's subjective pain, we used a VAS measurement tools. We found that inhalation of eucalyptus oil significantly decreased VAS pain scores compared with our control group. The major component of eucalyptus oil is 1,8-cineole, which had a morphine-like effect relieving pain in mice [8]. Another study also found that 1,8-cineole exhibited antinociceptive properties in rats and mice [15]. These findings, taken together with our results, suggest that subjective pain-reducing effects of eucalyptus oil are due, at least in part, to 1,8-cineole.

Serotonin has been considered to have an important role in the control of pain [16]. Activation of serotonin receptor presents on C-fibers has been shown mediating serotonin-induced pronociceptive effects [17]. Recent studies have shown that essential oils act via modulating of the central neurotransmitter system. *Hypericum perforatum* is considered to inhibit the synaptosomal uptake of serotonin [15]. Also, lemon oil reported to have an antixiolytic effects via the suppression of monoamines dopamine and enhancing serotonergic neurons [18]. Therefore pain-relieving effects of eucalyptus oil in the present results should be considered an involvement of serotonergic system. Pain and stress after TKR and during CPM are thought to act on the central and sympathetic nervous systems, increasing blood pressure and pulse. In the present study, group treated with eucalyptus oil inhalations showed statistically significant reduction in blood pressure, suggesting that eucalyptus oil could promote relaxation by reducing sympathetic activity while augmenting parasympathetic during CPM after TKR. A possible pathway for this explanation may include the olfactory system. Autonomic nervous system is affected by odorants, thus inhalation of essential oil is direct actions on the autonomic nervous system via olfactory system [19, 20]. Recent study has shown that olfactory stimulation with lavender essential oil and its active component, linalool reduced the sympathetic nerve activity in rats [21]. Therefore, eucalyptus oil also may modulate autonomic responses such as blood pressure via central nervous system and autonomic nervous system. The cardiovascular effects of 1,8-cineole were also investigated in several research. 1,8-cineole decrease blood pressure in normotensive rate and elicited a endothelium-dependent vasorelaxation in rate aorta [22, 23]. Other reports have shown that the administration of 1,8-cineole reduces contractile activity in rat [24]. Thus, there is a possibility that 1,8-cineole contributes to hypotensive effects of eucalyptus essential oil.

Eucalyptus oil has been used to treat influenza infection, owing to its anti-inflammatory and antibacterial effects [25]. Moreover, 1,8-cineole was found to reduce cytokines that cause inflammation in guinea pigs [20], and to significantly reduce edema and CRP at sites of inflammation throughout the entire body [26]. These studies prompted us to hypothesize that eucalyptus oil would reduce pain following TKR by lessening inflammatory reactions. However, markers of inflammatory responses, such as CRP concentration and WBC count, did not differ significantly between the our eucalyptus oil and control groups although comparisons before and after inhalation showed that the WBC count tended to be lower in the eucalyptus than in the control group, which may have been due to the time of inspection, the concentration of eucalyptus oil inhaled, and/or its frequency of application.

In summary, this study, which investigated the effects of eucalyptus oil inhalation on patients who underwent TKR, showed that eucalyptus oil inhalation was effective in reducing patient's subjective pain and blood pressure after surgery. These findings suggest that the inhalation of eucalyptus oil might be a valuable nursing intervention for pain relief after TKR.

## Figures and Tables

**Table 1:** Chemical compositions of the eucalyptus oil determined by GC-MS.

RT^a^ (min)	KI^b^	Compound	Area %^c^
12.952	1038	*α*-pinene	4.95
14.513	1075	*α*-fenchene	0.01
14.897	1084	Camphene	0.03
16.826	1128	β-pinene	0.75
17.571	1144	o-cymene	0.01
19.118	1177	b-myrcene	0.95
19.477	1184	*α*-phellandrene	1.09
20.221	1199	*α*-terpinene	0.24
21.447	1227	Limonene	13.68
21.985	1239	1,8-cineole	61.46
22.560	1251	trans-β-ocimene	0.33
23.431	1269	*γ*-terpinene	5.87
24.619	1293	*ρ*-cymene	8.55
25.152	1304	*α*-terpinolen	0.41
25.308	1307	Isoamyl isovalerate	0.04
28.679	1381	o-menthone	0.05
29.771	1403	*α*-pinene epoxide	0.02
32.286	1461	Dehydro-*ρ*-cymene	0.02
33.529	1489	cis-Limonene oxide	0.05
36.294	1554	Linalool	0.20
38.286	1600	Fenchyl alcohol	0.07
39.233	1624	4-Terpineol	0.38
41.487	1680	trans-pinocarveol	0.21
42.519	1705	cis-Citral	0.03
42.713	1710	Crypton	0.01
42.883	1715	*α*-terpineol	0.53
43.115	1721	*α*-terpinyl acetate	0.01
43.204	1723	Borneol	0.02
44.984	1770	Carvone	0.01
48.033	1852	cis-Carveol	0.02
48.139	1855	trans-Geraniol	0.004

^a^RT: retention time. ^b^KI: Kovats indices. ^c^Calculated with peak area obtained from GC/MS.

**Table 2:** General characteristics of the eucalyptus oil and control groups (*N* = 52).

Characteristics	Total (*n* = 52)	Control (*n* = 27)	Eucalyptus oil (*n* = 25)	*t* or *χ*²	*P*-value
Age (years)	68.2 (7.6)	67.5 (8.9)	68.9 (6.1)	0.69	.49^a^
Gender					
Female	48 (92.3)	24 (88.9)	24 (96)	0.19	.61^b^
Male	4 (7.7)	3 (11.1)	1 (4.0)		
Surgical classification					
Unilateral	37 (71.2)	18 (66.7)	19 (76.0)	0.19	.66^c^
Bilateral	15 (28.9)	9 (33.3)	6 (24.0)		
BMI (kg/m^2^)	26.4 (3.1)	26.1 (3.23)	26.7 (3.0)	−0.68	.50^a^
Education					
≤Elementary	34 (65.4)	16 (59.3)	18 (72.0)	2.17	.37^b^
Middle	5 (9.6)	2 (7.4)	3 (12)		
≥High	13 (25.0)	9 (33.3)	4 (16.0)		
Marital status					
Married	52 (100)	27 (100)	25 (100)		
Employed					
Yes	12 (23.1)	7 (25.9)	5 (20.0)	0.03	.86^c^
No	40 (76.9)	20 (74.1)	20 (80.0)		
Duration of osteoarthritis (years)	7.2 (5.5)	6.0 (5.2)	8.5 (5.7)	−1.67	.11^a^
Hypertension medication					
Yes	38 (73.1)	19 (70.4)	19 (76)	0.02	.89^c^
No	14 (26.9)	8 (29.6)	6 (24)		
Diabetes medication					
Yes	15 (28.9)	8 (29.6)	7 (28)	0.00	1.00^c^
No	37 (71.2)	19 (70.4)	18 (72)		

Data reported as mean (SD) or *n* (%).

Abbreviations: SD: standard deviation, BMI: body mass index.

^a^ *t*-test.

^b^Fisher's exact test.

^c^Chi-square test.

**Table 3:** Outcomes in the eucalyptus oil and control groups (*N* = 52).

Variables	Control (*n* = 27)	Eucalyptus oil (*n* = 25)	*P*-value
VAS (score)	5.0 ± 1.0	5.0 ± 0.0	.44
sBP (mm Hg)	120.0 ± 20.0	120.0 ± 20.0	.31
dBP (mm Hg)	80.0 ± 10.0	80.0 ± 10.0	.93
HR (beats/min)	78.0 ± 8.0	78.0 ± 10.0	.33
CRP (mg/L)	4.9 ± 10.5	7.2 ± 19.3	.59^a^
WBC (× 10^3^/*μ*L)	6793.3 ± 1396.6	6513.2 ± 2084.9	.57^a^

Abbreviations: VAS: visual analog scale; sBP: systolic blood pressure; dBP: diastolic blood pressure; HR: heart rate; CRP: C-reactive protein; WBC: white blood cell.

Wilcoxon's rank sum test. Data presented as median ± interquartile range.

^a^ *t*-test. Data presented as mean ± standard deviation.
