# Correction to “Effect of Eucalyptus Oil Inhalation on Pain and Inflammatory Responses after Total Knee Replacement: A Randomized Clinical Trial”

**DOI:** 10.1155/ecam/9765463

**Published:** 2026-05-30

Y. S. Jun, P. Kang, S. S. Min, J.‐M. Lee, H.‐K. Kim, and G. H. Seol, “Effect of Eucalyptus Oil Inhalation on Pain and Inflammatory Responses after Total Knee Replacement: A Randomized Clinical Trial,” *Evidence-Based Complementary and Alternative Medicine* 2013, no. 1 (2013): 502727, https://doi.org/10.1155/2013/502727.

In the above article, the data presented in Table 3 were incorrectly entered during manuscript preparation. The correct Table 3 is as follows:

**TABLE 3:** Baseline measurements before intervention in the control and eucalyptus oil groups (*N* = 52).

Variables	Control (*n* = 27)	Eucalyptus oil (*n* = 25)	*p* value
VAS (score)	5.0 (5.0–6.0)	5.0 (5.0–5.0)	0.44^a^
sBP (mmHg)	118.1 ± 11.1	122.4 ± 14.5	0.24^b^
dBP (mmHg)	74.8 ± 7.5	74.8 ± 7.1	0.99^b^
HR (beats/min)	77.3 ± 5.9	81.1 ± 12.0	0.15^b^
CRP (mg/L)	2.2 (0.9–3.9)	1.8 (0.7–2.1)	0.49^a^
WBC (× 10^3^/*μ*L)	7.0 (5.8–7.8)	5.8 (5.3–7.4)	0.26^a^

*Note:* VAS scores were recorded as whole‐number (integer) ratings on a 0–10 scale (no decimal values recorded).

Abbreviations: CRP, C‐reactive protein; dBP, diastolic blood pressure; HR, heart rate; sBP, systolic blood pressure; VAS, visual analog scale; WBC, white blood cell.

^a^Mann–Whitney *U* test; data presented as median (interquartile range).

^b^Welch’s *t*‐test; data presented as mean ± standard deviation.

Furthermore, the units for “WBC” in Figure 2(b) are incorrect. The correct Figure 2 is as follows:

**FIGURE 2:**
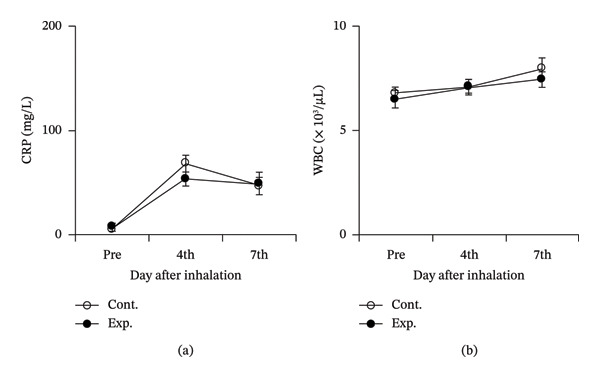
Effects of inhalation on (a) CRP and (b) WBC in the eucalyptus oil (*n* = 25) and control (almond oil; *n* = 27) groups. Values are expressed as mean ± SEM. Abbreviations: CRP, C‐reactive protein; WBC, white blood cell.

The corrections are limited to data presentation and do not affect the scientific conclusions of the study.

We apologize for these errors.

